# Collaborative Innovation and Absorptive Capacity as an Antecedent on IT Firm Financial Performance

**DOI:** 10.1007/s13132-023-01202-2

**Published:** 2023-05-04

**Authors:** Mário Nuno Mata, José Moleiro Martins, Pedro Leite Inácio

**Affiliations:** 1 ISCAL-Instituto Superior de Contabilidade e Administração de Lisboa, Instituto Politécnico de Lisboa, Avenida Miguel Bombarda 20, 1069-035 Lisbon, Portugal; 2 ISCTE-Instituto Universitário de Lisboa (ISCTE-IUL), Business Research Unit (BRU-IUL), Lisbon, Portugal

**Keywords:** Collaborative innovation, Financial performance, Absorptive capacity, Intellectual capital

## Abstract

A lack of collaborative innovation and absorptive capacity in firms causes projects to fail. Managers/employees in small and medium enterprises (SMEs) are not sufficiently aware of the practices of intellectual capital and nor do they collect, share, transfer, and utilize knowledge properly. This current study, therefore, focuses on the relationship between collaborative innovation and the financial performance of Portuguese IT sector SMEs, with a mediating role of absorptive capacity and a moderating role of intellectual capital based on three sub-domains (human capital, organizational capital, and social capital). Close-ended questionnaires were used to obtain data from 308 employees and managers. Owing to the COVID-19 pandemic, data were also collected through an online survey method. The simple random sampling technique was used to collect data and analyze it using the PLS-SEM method. The results show that collaborative innovation has a positive and significant impact on the financial performance of IT firms in Portugal. Absorptive capacity is considered a potential mediator between collaborative innovation and financial performance. Moreover, the moderating role of intellectual capital strengthens the relationship between collaborative innovation and absorptive capacity.

## Introduction

Innovation turns out to be unpredictable to such an extent that it cannot be overseen by a single organization (Lo et al., 2020; Mahmood & Mubarik, 2020). Collaborative innovation (CI) is a trans-disciplinary approach used to increase overall cooperation in order to further the effectiveness of associating side-to-side, rounded, reasonable, and consistent relations among revolutionary contributors in a positive atmosphere (Stojčić, 2020). Information technology (IT) firms, especially small and medium enterprises (SMEs), are turning to new ways to open up to overcome their skills shortages (Benhayoun, Le Dain, et al., 2020; Thomas et al., 2021). In fact, while SMEs contribute to the promotion of innovation due to their inventiveness, ingenuity, and market approach (Kraus et al., 2020), they consolidate external resources to remain competitive and help maintain high levels of internal performance within a limited number of technology fields (Ran et al., 2021). To acquire valuable innovation skills, SMEs rely heavily on collaborative strategies (Carrasco-Carvajal et al., 2022). In this regard, they are developing new CI involving various actors working together in a state of mutual trust and strong exchanges to achieve a mutually beneficial goal (Hong et al., 2019).

The resource-based view (RBV) theory advises that immaterial assets, like essential intelligence, are the foremost motivating services after organizational competitive advantage (Lichtenthaler, 2016). According to Barney (1996), RBT treats initiatives as latent inventors of value-added competences. Empathetic, the improvement and immersion of such aptitudes, and the primary administrative abilities, contain inspecting the properties and properties of the well-founded from a scholarly capital perception (Cheah & Yuen-Ping, 2021). There has already been considerable exploration of the applications, reproductions, and tactics for CI and the petition for refining the presentation of CI and importance of CI for financial performance (FP) (Benhayoun et al., 2020; Feranita, 2021; Wang et al., 2017). Around is, nevertheless, a deficiency of a methodical study of such a gentle buttressed by experimental educations from the standpoint of absorptive capacity (Feranita et al., 2017). The idea of the absorptive capacity (AC) of the project supervisor has advanced and extended from a static view, which centers on earlier learning, to a progressively powerful, process-based point of view that underscores aggregate capacity (Ávila, 2021). Project-based IT organizations ordinarily need to create absorptive capacity in order to keep up their productive performance in the global market (Bolívar-Ramos et al., 2013). They are compelled to team up with different firms, networks, and, specifically, colleges to separate their item contributions from those of universal contenders with lower production. Firms enriched with more noteworthy absorptive capacity of undertaking administrator relied upon to beat rivals.

Surprisingly, one of the widespread strategies used for refining the performance of collaborative innovation in management to improve financial performance relies on intellectual capital (Mutuc & Cabrilo, 2022). Progress in developing innovation depends on intellectual capital and how it is designed, shared, and immersed in information technology production. There is widespread acknowledgement that control of intellectual capital is crucial to the smooth running of any type of business (Chernenko et al., 2021; Weqar et al., 2021). The acceleration of technological progress, particularly in the area of information technology (IT), requires the adoption of more effective methods by organizations to accept and implement innovative technologies. However, previous research in this field is limited in scope and often outdated, failing to keep up with the ever-changing dynamics of IT usage and adoption. Therefore, a comprehensive reevaluation and refinement of existing approaches to managing IT innovation are imperative for organizations to keep pace with the constantly evolving technological landscape and remain competitive.

## Literature Review

### Impact of Collaborative Innovation on Financial Performance

The capacity to properly handle today’s business climate is just one component in gaining a competitive advantage. More importantly, the company’s ability for innovation decides how far those regulations may be modified (Kamboj et al., 2015). The process of transforming a unique idea into a profitable product or service that people may buy is known as innovation. Innovation through collaboration based on the RBV, companies form partnerships to get access to shared resources, with the goal of encouraging innovation through the exchange of valuable expertise (Barney, 1991; Cheng et al., 2022; Stojčić, 2020). Using RBV as a theory, Thomas et al. (2021) found that businesses collaboration influence was motivated by their strategic positioning in the market and their innovation plans. Surprisingly, there is a limited level of collaboration among growth-stage businesses. That is probably because they do not have the resources to attract collaborative partners and have not developed the capacity to innovate yet. Although Manik and Lukito-Budi (2020) study on collaboration from an RBV viewpoint has been essential, it only focuses on some facets of RBV. Recent research by Kamboj et al. (2015), which incorporates RBV, provides more insight into how various types of business resources influence the likelihood of forming partnerships.

Therefore, the literature on collaborative creativity is vast, including a wide range of perspectives from many schools of thought and dispersed across all types of enterprises and countries (Agger & Lund, 2017). While there may be internal or external collaborative innovation, current study focuses on collaborative innovation influence on IT firms’ financial performance. Tactical collaborative innovation, transformative collaborative innovation, and collaborative partnerships are all distinct aspects of collaborative innovation (Southern, 2005). Tactical collaborative innovation refers to a company’s goal of superiority through immediate opportunity-seeking and advantage-seeking actions (Ind et al., 2017). Both small and large businesses encounter challenges in their goal of tactical collaborative innovation. SMEs’ opportunity-seeking competence may be strong, because their limited knowledge share and lack of market power limit their capacity to execute the competitive benefits required to extract appropriate value from the opportunities they choose to explore (Thomas et al., 2021).

Large enterprises, on the other hand, are experts at establishing competitive advantages, but their significant focus on the competency of their existing operations often undermines their capacity to continually explore further chances (Kraus et al., 2020). Collaborative innovation is a strategy for increasing business capacity, encouraging team members to engage in decision-making, and using each partner’s knowledge as key components (Ran et al., 2021). This style of teamwork has the power to transform individuals as well as businesses (De Jong & Freel, 2010). Furthermore, collaborative innovation may establish knowledge for revolution; we know a little bit about how these productive innovation evolved (Anderson & Hardwick, 2017; Cricelli et al., 2021). It is suggested that collaborative innovation may recognize IT firms in their particular issues, based on a number of ideas such as network and resource-based innovation.

Collaborative innovation is focused on openness, trust, and benefits’ sharing. Companies work closely with their internal and external stakeholders over the long term. By collaborating with its supply chain partners, a company may better allocate its resources and save its expenses. More businesses are trying to implement collaborative innovation as a response to increased competition, shorter product life cycles, and a broader range of available markets. Joint innovation efforts in the supply chain that are aimed on satisfying customer demand are a primary emphasis of this kind of collaboration. It enables businesses all throughout the supply chain to use the benefits of IT to boost delivery performance and secure long-term competitive advantages. It has been shown that collaborative innovations are important to wealth creation in IT sectors and contribute significantly to the financial performance of businesses (Mondal & Ghosh, 2012; Un et al., 2010). Typically, IT firms’ financial performance has been elevated by a combination of profitability ratios, benchmarking, calculating recital against financial plan, or a combination of these strategies (Rosita et al., 2020). Earlier research has proven a long-term relationship between innovation and performance (e.g., (Perez-Luno et al., 2014; Petrakis et al., 2015; Thomas et al., 2021). Therefore, we propose our first hypothesis:

H1: Collaborative innovation is positively associated with financial performance.

### Collaborative Innovation and Absorptive Capacity

The IT sector negotiates the product-service field and is occupied by administrations that convey nonspecific and modified software, technology structure, technical sustenance, and consultancy. These administrations are significant for co-designing products and facilities for outside clients and scheming new organisms for active use by inner clients (Hong et al., 2019). An IT organization is capable to structure and manage its innovation process using an intellectual capability and absorptive capacity. Collaborative innovation is conventional on shared trust, frankness, danger, and advantage involvement (Najafi-Tavani et al., 2018). Administrations launch long tenure and close collaboration affiliations with contractors and clients. Each association shares statistics with other administrations in its source chain to enhance source allocation and reduce overall source series expenses, accordingly gaining modest benefits (Santoro et al., 2020). Market variation, value conflicts, and condensed product lifecycles have led extra administrations to struggle to accept collaborative innovation. Therefore, absorptive capacity is an organization’s ability to identify the value of new information, integrate it, and apply it to profitable ends (D’Angelo et al., 2020). It influenced by greatly on prior related information and variety of the background in administrations.

Absorptive capacity is accumulative, sense that it is calmer for an association to capitalize on a continuous basis in its absorptive capability than investing promptly (Apa et al., 2020). Kostopoulos et al. (2011) suggested that absorptive capacity is a set of managerial routines and methods, by which administrations obtain, integrate, transform, and exploit information to create a vibrant structural capability. Mahmood and Mubarik (2020) states that the absorptive capacity is a significant source for the association performance. The concept of potential absorptive capacity is planned in which the attention is more on knowledgeable capability and assimilation capability. It can be distributed into demand-pulling capability and technology-driving capability (Murovec & Prodan, 2009). There are several precarious aspects that affect absorption capacity of administrations containing inner exploration and progress, personnel activity, innovative collaboration, and initiative approach (Miroshnychenko et al., 2020). Collaborative innovation cannot only speed up the flow of information and understanding between administrations, but also improve administrations’ information accumulation and form actual learning and communication appliances, thus encouraging organizational absorptive capacity (Manik & Lukito-Budi, 2020). In organizations, there is a well-recognized connection between collaborative innovation and absorptive capacity:

H2: Collaborative innovation is positively associated with absorptive capacity.

### Absorptive Capacity and Financial Performance

An organization’s absorptive capacity is described as its ability to perceive, integrate, acquire, transform, and exploit new knowledge (De Jong & Freel, 2010). This definition emphasizes the diversity of individuals in the workplace regarding their ability to comprehend new knowledge, change its meaning, integrate it into the organization, and eventually allocate it for use and application (Zhang et al., 2021). Individual integration occurs due to analyzing and using project information (Flatten et al., 2011; Papazoglou & Spanos, 2021). In the current period of development, there has been a noticeable movement in organizations in which the competencies of individuals in workgroups are seen as one of the most critical factors for increasing creativity, learning lessons, and financial performance (Manik & Lukito-Budi, 2020; Yafi et al., 2021). Individuals that are diverse in their knowledge backgrounds are more likely to integrate and absorb from one another, resulting in enhanced creativity, innovation, and, as a result, financial performance (Haider & Kayani, 2020; Miroshnychenko et al., 2020). Thus, we hypothesize that after a fact, increases in absorptive capacity will be counterproductive to additions in firm financial recital. The above literature leads to hypothesize that:

H3: Absorptive capacity is positively associated with financial performance.

### Absorptive Capacity as a Mediator Between Collaborative Innovation and Financial Performance

The high level of collaborative innovation has increased the frequency of direct communication between the company’s leading players and its external players (Hong et al., 2019). As a result, organizations that collaborate with external parties are better position to innovate because they have more access to the resources needed to develop new goods and processes. While coordinated effort with market actors can assume a vital part in firms’ advancement abilities. Therefore, absorptive capacity has been regarded as crucial to maintaining a competitive environment (Chaudhary & Batra, 2018). When an organization’s knowledge-based assets are redefined and skillfully structured, an organization can handle changes in a convenient and sensitive manner. Through its capabilities, it will boost its growth, align the change with activity and other domains, and thus enhance its innovation and financial performance (Murovec & Prodan, 2009; Papazoglou & Spanos, 2021). Based on the competitive view of potential, companies with high absorptive capacity are likely either from rivals, consumers, channel partners, and suppliers to gain new expertise externally. Such know-how is used in businesses to recognize business opportunities such as consumers push, technological advancement, an unpredictable world, and the trending to changing the market places (Benhayoun, Le Dain, et al., 2020), all of which would increase the level of profit and market share significantly (D’Angelo et al., 2020). In addition to commercially relevant practical applications, absorptive capacity can incorporate new external expertise, create beneficial prospects, and improve productivity (Cheah & Yuen-Ping, 2021). As regards quality and value, absorptive capacity provides a margin for growing business overall growth. The fourth hypothesis is therefore formulated as follows:

H4: Absorptive capacity as a mediator between collaborative Innovation and financial performance.

### Intellectual Capital as a Moderator Between Collaborative Innovation and Intellectual Capital

Intellectual capital is a collection of immaterial resources used in organizations to generate added value (Khalique et al., 2015). IC consists of three sub-dimensions: human capital, organizational capital, and social capital (Baima et al., 2020; Bontis et al., 2015). The highest and most valuable intangible resource of the business is human capital. It contains knowledge, experience, talents, and skills shared within the organization. Human capital (HC) includes the expertise, preparation, knowledge, and expertise of its members (Chernenko et al., 2021; Denizci & Tasci, 2010). Financial performance is a method to grow intellectual capital that has been tailored to meet the organizations’ needs to produce intellectual capital (Kianto et al., 2017). Selected workers increase human resource efficiency by ensuring that professional and experienced applicants are recruited (Gürlek & Tuna, 2019). Similar incentive programs are also powerful resources to maintain the enterprise and recruit skilled human capital. Furthermore, social capital (SC) means social norms, beliefs, principles, ties, friends, trusts, responsibilities, flows for knowledge, social norms, mutual benefit commitments, collective acts, and social and economic development contributions (Xu & Liu, 2021). SC is called networks of connections between people who live and work in a community to make them more successful (Gupta & Raman, 2021). The SC definition was initially known in community studies and was used to describe one person’s connection to the other in the community (Chang et al., 2006).

Considering the characteristics of small- and medium-sized enterprises (Berends et al., 2014), it should be added that their material assets and finances are small, which means that their competitive advantage will be derived mainly from intangibles (Silva & Moreira, 2021). Also, small business owners do not have access to a large number of important market studies and data or do not have a proper control system in place (Kraus et al., 2021). This makes it very important for an organization’s finances to reach the right performance level that focuses on how organizations are structured, the processes that they use, and the systems they use. Lastly, organizational capital (OC) incorporates activities that impact company performance and is related to priorities and strategic planning, preparation, job descriptions, coordination, and decision-making communication for employees (Dženopoljac et al., 2016; Haider & Tehseen, 2022). This definition is clear but strategically distinct, according to Martín‐de‐Castro et al. (2006), as workers own the human capital, these organizations, including organizational culture, technical processes, and formal structures, own and maintain organizational capital, all of which help organizations absorb and refine understanding.

**Fig. 1:**
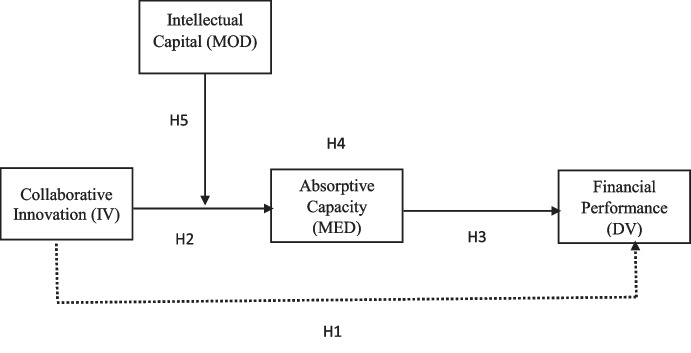
Conceptual model

Resource-based view theory adds to its external trade situation (what advertise requests and what contenders offer) a firm inner potential. The capacity to clarify and call intangible or moderate materials frequently, Amit and Schoemaker (1993) have proven more resilient. The internal capacity essentially refers to the ability of the organization to prepare capital. The main asset which sees such benchmarks is the “learning” of whether they are referred to as unrecognizable resources, absorbent skills, central skills, essential resources, incorporeal resources, structure memory, or similar alternative ideas. The study found a positive correlation between collaborative innovation and the company’s absorptive capacity. Absorptive capacity shows the economy to take advantage of the knowledge and incorporate it for ultimate purposes (Soo et al., 2017). Zahra and George (2002) employed it in an organizational context, describing AC as an organization’s ability to recognize, integrate, and implement the value of the latest information for commercial ends. Therefore, based on arguments above, the following hypothesis is established (Fig. 1):

H5: Intellectual capital as a moderator between collaborative innovation and absorptive capacity.

## Research Methods

### Sample and Data Collection

The current research purpose was to inspect the effect of collaborative innovation on firm performance of Portugal. The study is based empirically on a sample of small- and medium-sized enterprises operating in the Portuguese due to COVID-19 pandemic; there is rapid growth seen in IT SMEs as shown in the Organisation for Economic Co-operation and Development (OECD) report 2020 (Lamichhane et al., 2021). For the current study, simple random sampling technique was used, and data were obtained from both public and private project-based software organizations operating in different cities of Portugal from November 2020 to February 2021. Data has been collected through a self-administered paper-and-pencil survey and in some cases through online survey due to COVID-19 pandemic. The respondents are programming managers, designers, project supervisors, and operation managers working in different IT SMEs companies. The G*Power software shows 119 respondents to get a capacity of 0.95 and a medium impact of 0.15 for this study as the minimum sample size (Hair Jr et al., 2016). Scholars received data from 335 target respondents, which was more than the minimum sample size needed. Initially, 400 questionnaires were distributed, and 7 questionnaire was excluded based on missing information and incorrect answers, which make these questionnaires inconclusive and thus excluded. However, only 308 usable surveys were received, resulting in a 77% response rate.

The questionnaire consisted of two areas. Section A covered personal information such as age, gender, marital status, job title, and experience. Section B included questions to evaluate the link between independent and reliable diversity. Males made up 52.7% of the 308 respondents, while females made up 47.3%. Most of the participants had bachelor’s and post-graduate degree holder and age between 20 and 40 years. The strength shows that most respondents had experience in projects between 1–5 years and above 6 years. After data collection, Harman’s one-factor analysis findings showed that the study had no problems with common method analysis, based on the exploratory factor analysis and the principal analysis approaches, since the single factor explained a cumulative variance of 19.33%, lower than the suggested ·50% threshold (Tehseen et al., 2017).

### Measures

This research follows a deductive approach, seeking to understand causal connections between the selected variables. Evaluating the absorptive capacity as mediator between collaborative innovation and financial performance leads to the creation of a range of hypotheses also measure the role of intellectual capital as a moderating variable of second order in the three dimensions: human capital, organizational capital, and social capital. All items have been evaluated at a 5-point Likert scale. This study consists of a closed ended questionnaire from various sources used to evaluate four variables. An 11-item questionnaire is adapted for collaborative innovation by Bucic and Ngo (2012). To measure financial performance 4-item scale created by Shashi et al. (2019) was embraced. The 10-item scale mediating variable absorptive capacity was used developed by Zahra and George (2002) and moderating intellectual capital as second-order variable based on 14-item scale (human capital (5 items), organizational capital (4 items), and social capital (5 items)) adopted from Singh and Rao (2016).

## Results

### Analytical Technique

The analysis was applied using Smart PLS tools v.3.0 to measure structural equation modeling (SEM) (Hair et al., 2019). SEM is a multidisciplinary approach used commonly to research relationships with structures (Mai et al., 2021). It allows the simultaneous analysis of multiple variables in an integrated model (Hair Jr et al., 2016). Due to the limited sample and non-parametric nature of the results, PLS was favored over other techniques. Relatively low sample size can be determined by PLS-SEM. This method is equally successful for the study of non-distributed data (Hair et al., 2017).

### Validity and Reliability

**Fig. 2:**
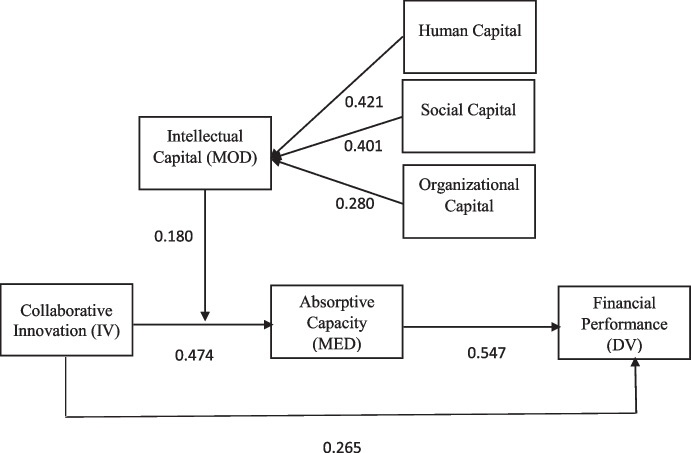
Measurement model analysis

The adoption of the PLS-SEM approach for data analysis includes an evaluation of efficiency and adequacy of structural models based on measuring parameters that analyze the reliability and validity of the model (Henseler et al., 2015; Mai et al., 2021). The bootstrapping method (5000 sub-sample for *t* test) was used to assess load, weight, and path coefficients for 308 cases (Hair et al., 2017). Table 1 displays the effects of the validity and reliability controls. The values for composite reliability (CR) and Cronbach’s alpha (α) were tested for internal consistency. Table 1 findings demonstrate the buildings’ internal consistency: values are greater than 0.70 for CR and for the of Cronbach’s. Also, factor loading values were evaluated to verify the reliability of indicators. Factor loading should exceed 0.70, according to Hair et al. (2017), to assess the reliability of the indicator. The average variance extracted (AVE) values should be greater than the specified threshold of 0.50. However, some indicators from CI6, CI9, CI10, and CI11 items were removed in order to boost the value of AVE. Hair et al. (2017) introduced this strategy after discovering that items with loadings between 0.40 and 0.70 should be eliminated from deleting the variable observed will improve the reflective scale composite reliability. Accordingly, after the removal, all estimations of factor loadings, CR, and AVE are greater than the suggested cut off criteria; therefore, Fig. 2 states that, since all thresholds are met, the model and its constructions are internally consistent and convergent invalidity.

**Table 1:** Measurement model

Constructs/items	Items	Factor loadings	α	CR	AVE	Authors
Absorptive capacity			0.892	0.909	0.504	Zahra and George (2002)
We are successful in learning new things within this group	AC1	0.764				
We are effective in developing new knowledge or insights that have the potential to influence product development	AC2	0.790				
We have effective routines to identify, value, and import new information and knowledge	AC3	0.792				
We have adequate routines to analyze the information and knowledge obtain	AC4	0.805				
We have adequate routines to assimilate new information and knowledge	AC5	0.826				
We are effective in transforming existing information into new knowledge	AC6	0.592				
We can successfully exploit internal and external information and knowledge into concrete applications	AC7	0.628				
We are effective in utilizing knowledge into new products	AC8	0.644				
We are able to identify and acquire internal (e.g., within the group) and external (e.g., market) knowledge	AC9	0.590				
Prior to the project, did your project team have the expertise required to assimilate the knowledge that came from the other subsidiaries?	AC10	0.606				
Collaborative innovation			0.834	0.880	0.524	Bucic and Ngo (2012)
New product prototypes (still in the development stage)	CI1	0.804				
New products or services introduced to the market which are new to the market or the firm	CI2	0.811				
Significant modification to existing products or services	CI3	0.793				
New/modified production or manufacturing techniques	CI4	0.791				
New/modified administration or managerial techniques/practices/policies	CI5	0.824				
New/modified marketing (inc advertising and distribution) techniques	CI7	0.466				
Patents either applied for, pending or obtained	CI8	0.460				
Financial performance			0.866	0.911	0.720	Shashi et al. (2019)
The return on investment of our company is higher compared to competitors	FP1	0.921				
The return on assets of our company is higher compared to competitors	FP2	0.801				
The sales growth and profitability of our company are higher compared to competitors	FP3	0.730				
The total operating costs of our company are lower compared to competitors	FP4	0.925				
Construct: intellectual capital *Sub-construct*						Singh and Rao (2016)
Human capital			0.850	0.893	0.626	
Employees are highly skilled	HC1	0.781				
Employees are widely considered the best in our industry	HC2	0.774				
Employees are creative and bright	HC3	0.829				
Employees are experts in their particular jobs and functions	HC4	0.809				
Employees develop new ideas and knowledge	HC5	0.762				
Organizational capital			0.825	0.884	0.656	
Organization uses patents and licenses as a way to store knowledge	OC1	0.826				
Organizational knowledge is contained in manuals, databases, etc.	OC2	0.773				
Organization’s culture (stories, rituals) contains valuable ideas, ways of doing business, etc.	OC3	0.821				
Organization embeds much of its knowledge and information in structures, systems, and processes	OC4	0.819				
Social capital			0.837	0.885	0.609	
Employees are skilled at collaborating with each other to diagnose and solve problems	SC1	0.810				
Employees share information and learn from one another	SC2	0.838				
Employees interact and exchange ideas with people from different areas of the company	SC3	0.817				
Employees interact with customers, suppliers, alliance partners, etc., to develop solutions	SC4	0.780				
Employees apply knowledge from one area of the company to problems and opportunities that arise in another	SC5	0.641				

Abbreviations: *CR* composite reliability; *α* Cronbach’s alpha, *AVE* average variance extracted

### Discriminant Validity by Fornell–Larcker Criterion

After confirming that the model has converged and fulfilled the pre-set criteria, the next step was to validate the model discriminatory by Fornell–Larcker criterion. Fornell–Larcker states that the square root of the AVEs should be greater than the construct’s correlations (Fornell & Larcker, 1981). Table 2 shows a discriminating validity since the square roots of AVEs are greater than the correlations between structures, as shown by the bold products.

**Table 2:** Discriminant validity by Fornell–Larcker criterion

Constructs	1	2	3	4	5	6	7
1. Absorptive capacity	0.788						
2. Collaborative innovation	0.710	0.724					
3. Financial performance	0.759	0.710	0.848				
4. Human capital	0.520	0.503	0.481	0.791			
5. Intellectual capital	0.497	0.470	0.465	0.716	0.944		
6. Organizational capital	0.309	0.283	0.316	0.641	0.809	0.810	
7. Social capital	0.483	0.452	0.436	0.864	0.780	0.631	0.939

### Assessment of Second-Order Construct

After analyzing and validating the first-order constructions, the second-order construction for multicollinearity of items and analysis of outer weight and its importance was reviewed. A two-stage process was presented to analyze the second order (Hair et al., 2017). First, the latent variable values of the lower-order components were determined. Intellectual capital scores are used for all variables after the latent variables have been determined in the initial step. The intellectual capital measurement approach was evaluated based on Hair et al. (2017), and the results are reported in Table 3. The variance inflation factor (VIF) and a high correlation between two or more construct elements are used to achieve multicollinearity (Hair et al., 2017). The reflective construct was investigated in multicollinearity. A number greater than 5 indicates multicollinearity. Table 3 shows that multicollinearity is not a concern based on the second-order reflective, dimensional VIF values. The reflective indicators’ outer weights were evaluated. Bootstrapping also checked the weights’ value. The importance and weight of the measures are depicted in Fig. 2. External human capital, organizational capital, and social capital weights were all relevant for one item, as shown in Table 3.

**Table 3:** Outer weights and VIF values

Relationship among constructs	Original sample	Sample mean	Standard deviation	*t* statistics	VIF	LLCI 5.0%	ULCI 95.0%
Human capital -> intellectual capital	0.421	0.421	0.010	40.521	3.184	0.402	0.443
Organizational capital -> intellectual capital	0.280	0.279	0.012	23.135	1.768	0.255	0.302
Social capital -> intellectual capital	0.401	0.401	0.010	39.175	3.096	0.381	0.421

### Structural Equation Model

**Fig. 3:**
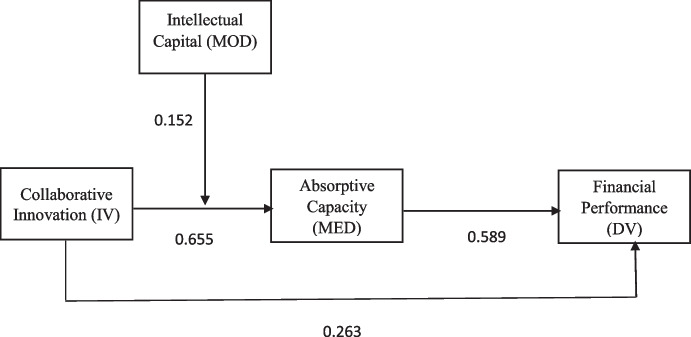
PLS path analysis of *n* = 5000 bootstrapped samples

The structural equation model is calculated after the measuring model is finished. This study used the standard bootstrapping procedure to obtain a significant level of any link between the constructs. To investigate the mediating effects of absorptive capacity, we use the methodologies proposed by Henseler et al. (2015). The direct and indirect effects of the structural equation models were assessed using four key parameters: to begin, determine the sum of variance explained by all constructs in endogenous latent variables *R*^2^ (Hair et al., 2018). Although an adequate evaluation of *R*^2^ relies on the analysis (Cohen, 1988), there is a high, moderate, and low evaluation of 0.26, 0.13, and 0.09, respectively. In the current study, however, *R*^2^ values for the endogenous variable financial performance are anticipated to be 65.1% due to collaborative innovation and absorptive capacity. In addition, the predicted *R*^2^ for absorptive capacity for collaborative innovation and intellectual capital is 61.2%, and the model exhibits appropriate precision in prediction, as demonstrated in Table 4 and Fig. 3.

**Table 4:** Coefficient of determination

Constructs	*R* square	*R* square adjusted	*Q*^2^ (=1-SSE/SSO)
Absorptive capacity	0.612	0.608	0.298
Financial performance	0.651	0.649	0.460

Second, a cross-validation redundancy measure was also applied to determine predictive validity to estimate the study model has validated relevance (Hair Jr et al., 2016). In Table 4, the importance of the direct effect of a model can be observed since the model’s predictive importance is considered acceptable because *Q*^2^ values are more significant than zero (Henseler et al., 2015). The direct influence of collaborative innovation on financial performance (β = 0.262, *p* 0.001), collaborative innovation on absorptive capacity (β = 0.495, *p* 0.001), and absorptive capacity on financial performance (β = 0.562, *p* 0.001) is further supported by the H1, H2, and H3 results. As a result, each of the three direct hypotheses was accepted.

Third, effect size (*f*^2^) is an independent variable that expresses the external (independent) effect on the dependent variable (Hair Jr et al., 2016). The effect size values are 0.02, 0.15, and 0.35, respectively, according to the Cohen (1988), with small, medium, and significant effects. The CI to FP impact size is 0.084, the CI to AC effect size is 0.818, and the AC to PF effect size is 0.421, according to Table 5. The findings show that these exogenous components have a medium and high impact on the endogenous structure. Finally, for this analysis, the model suggested and validated that absorptive capacity can mediate the relationship between collaborative innovation and financial performance. Table 5 describes the lower limit confident interval (LLCI) and upper limit confident interval (ULCI) of .319 and .457. Both the ULCI and LLCI have the same sign positive, and there was no zero present between these two. Hence, we can conclude from here that mediation is happening. The significant and positive indirect effect from AC to FP (β = 0.387 and *p* < 0.05), as discussed in Table 5, is less than the direct effect. However, if the effect is indirect and significant but less than direct, it will also be shown to be partially mediated; the hypothesis 4 was therefore accepted.

**Table 5:** Structural equation model results

Hypothesis	Relationship between constructs	β	Mean	S.D.	*T* values	*f*^2^ values	LLCI 2.5%	ULCI 97.5%	Remarks
	Direct effects								
H1	CI -> FP	0.262***	0.261	0.049	5.350	0.084	0.168	0.356	Supported
H2	CI -> AC	0.655***	0.654	0.035	18.495	0.818	0.582	0.722	Supported
H3	AC -> FP	0.591***	0.592	0.047	12.507	0.421	0.498	0.681	Supported
	Indirect Effects								
H4	CI -> AC -> FP	0.387***	0.387	0.035	11.122		0.319	0.457	Supported
H5	CI * moderating effect 1 -> AC	0.152**	0.151	0.038	7.961		0.317	0.486	Supported

Abbreviations: *AC* absorptive capacity, *FP* financial performance, *CI* collaborative innovation, *IC* intellectual capital, *S.D.* standard deviation, *f*^2^ effect size, *LLCI* lower limit confident interval, *ULCI* upper limit confident interval *p<0.05, **p<0.01, ***p<0.001

Hypothesis 5 enunciated that intellectual capital moderates the relationship between collaborative innovation and absorptive capacity. The values in the Table 5 provided support for the hypothesis of moderation. The results showed that interaction term of “collaborative innovation and intellectual capital” moderates on the relationship of collaborative innovation and absorptive capacity both the LLCI=0.317 and ULCI=0.486 has the same sign, and there was no zero present. In addition, intellectual capital has a positive and significant moderating effect (β = 0.152, *p* <0.05). Therefore, the findings have demonstrated the support for hypotheses H4 and H5.

## Discussion

The objective of this study was to examine the impact of collaborative innovation on IT firm financial performance through mediating role of absorptive capacity and moderating role of intellectual capital. The study-based empirical analysis on a sample of SMEs operating in the Portuguese IT sector due to the COVID-19 pandemic rapid growth was seen in IT sector at SME level. This study looked at the complex role of absorptive capacity on the relationship between collaborative innovation and financial performance. The results also support the statement that collaborative innovation is a crucial catalyst for the capacity to absorptive. This means that knowledge acquisition, assimilation, change, and exploitation are made easier by capable workers, effective organizational structures, and good relations with stakeholders. This study demonstrates that intellectual capital has a positive moderating effect on collaborative innovation and absorptive capability, which is in line with previous research results from (Ávila, 2021; Engelman et al., 2017; Soo et al., 2017).

Additionally, the alteration of newly assimilated information is likely to not happen immediately or without struggle. Previous knowledge is essential to the firm’s capability to worth new information (Haider & Kayani, 2020). As companies attain information from beyond afield bases, it is less probable that the firm will own the preceding knowledge essential to fully figure out and properly value its findings, principal to wasted chances. Therefore, at extraordinary stages of absorptive capacity, previous information confines additional constrain the proficiency of information absorption efforts. Occupied together, the costs accompanying with the acquisition, integration, and alteration of new information would be estimated to overhaul the economic revenues connected with the manipulation of that information at high levels of absorptive capacity. Thus, we hypothesize that after a fact, increases in absorptive capacity will be counterproductive to additions in firm economic recital. Therefore, absorptive capacity is positively associated with financial performance.

However, previous studies have shown that collaborative innovation, absorptive capacity, and skills substantially affect financial performance. Companies are reluctant to invest in their employees. For various reasons, some scholars have identified for a long time, including that their organizations cannot wholly own individuals; many companies are also unable to invest financial resources in intellectual capital (Lyver & Lu, 2018; Rehman et al., 2018). Most of the studies identified positive relations that examined intellectual capital and absorption. Therefore, this study verified that intellectual capital is a good forecaster for absorptive capacity also for financial performance of SME’s.

## Theoretical and Practical Implications

The present study has provided a theoretical implication by giving further empirical evidence in the domain of resource-based theory, where collaborative innovation has been hypothesized as a resource to better understand the relationship among intellectual capital to attain and maintain absorptive capacity. The result showed that all IC dimensions have a direct influence on absorptive capacity; the willingness of a firm to learn external information will boost the output effect of IC. There is, however, a lack of awareness to grasp their integrated relations (Cricelli et al., 2021). This study helps to comprehend the value of AC to boost the efficiency of an organization. Moreover, AC helps the businesses to obtain and use external information effectively, improve their learning capacity, respond to environmental changes, and innovate. Furthermore, the intervention of an absorptive capacity system nevertheless significantly enhances the link between collaborative innovation and financial performance. It can thus be concluded that absorption of information by stakeholders and employee applicability affect company performance positively. This research support IT industry of Portugal to understand the importance of collaborative innovation in order to achieve financial objectives.

In the domain of project-based IT organizations, the findings of this investigation would give insights to SMEs which confront the trouble dealing with the intangible resources comparing to the globalization period of innovation and information-based economy (Mata et al., 2021). It would likewise help the Portuguese IT firm managers to evaluate the variables in for well financial performance (Martins et al., 2018). This study adds practically towards the business that in order to diminish the complexity, jointly efforts by whole team are necessary with excessive communication when situation is complex and in order to avoid misleading details, information sharing with joint decision-making strategy must be followed to let the team members leads towards success of project. The companies of Portugal have to spend more in intangible resources other than putting resources into old style factors of productivity. The organization has to invest more in intellectual capital and its components for the better productivity and profitability in the future.

## Limitations and Future Directions

As it is not possible to cover all aspects in one study, a few limitations are always there in research although these are tried to eliminate. A few research gaps have been filled by adding appreciative facts in literature. On the other hand, time and resource restrictions are some of the limitations associated with this study. The study is focused only on the project-based IT and SMEs of Portugal, and other sectors may not be generalized by the results. Future studies can test this model in other field, i.e., construction sector and hotel industry. The data collection for the present study is cross-sectional due to time and resource limitations; future research can consider conducting a study by utilizing longitudinal study as it helps in illustrating the causal relationship comprehensively. The model was analyzed by the single mediator and moderator; future researches can also focus on the mediating role of other variables between the relationship of collaborative innovation and financial performance, i.e., organizational ambidexterity and collaborative strategies. Also, future research may investigate the moderating effect of employee learning, social interaction on the relationship between collaborative innovation and absorptive capacity. With the addition of more relevant variables, the existing grounds for the research in this particular field can really be increased.
